# Mal-Nutrition,—Its Causes

**Published:** 1878-09

**Authors:** 


*Mal-Nutrition.—Its Causes.*—In the new edition of Ringer's Hand-book of Therapeutics, is a section on "Phosphate of Lime," which is suggestive, as bearing on the subject of mal-nutrition, its causes and associated conditions; and it is worth a careful study by those who fancy that lime-salts in deficient quantity is in any way associated with rachetis. Ringer truly says, "wherever cell growth is active, there is phosphate of lime in excess, and this holds good with regard to either healthy or diseased growths; for this salt is found to prevail in disease associated with rapid formation." Bearing in mind the fact that the intercellular fluid is acid and that phosphate of lime is acid, we see how reasonable it is to expect that this salt may be found in excess in this fluid; and bearing in mind, further, that their diffusive power is very small—that only a few grains pass into the blood, *i. e.*, of ammoniated phosphates—we see the weakness of a theory which expects nutrition from artificially ingested phosphates.

It is not a lack of phosphates, but a lack of the power to assimilate these; it is not lime-salts we need, but the power on the part of the system to take up and appropriate them, that the hard tissues lack development. There is an abundance of free and soluble phosphates of lime in the system of any child to make ten sets of teeth—indeed, as Ringer shows in defective nutrition the urine is loaded with phosphates, but that it is the *power of appropriation* that is wanting. If the teeth and tissues are starving, it is not for lack of proper food in the system, but from want of power in the system to work up these materials into hard structures. There is usually in all ordinary food abundance of phosphate of lime for the ordinary uses of the system, and if the teeth of our children suffer, we must look to some other means for a correction of the trouble.

It seems doubtful if phosphates, other than those incorporated by nature with our food, pass into the blood, since this artificial administration causes no augmentation of their salts in the urine; but however this may be, it is certain that phosphate of lime, as a therapeutic agent, is with difficulty assimilated, in spite of the fact that it is readily soluble in the free acids of the gastric juice. Just why phosphate of lime is useful (as Beneke shows) in those diseases in which the urine is loaded with it, is certainly a difficult question to decide ; and our knowledge is deficient here, as in many other directions.

Of the therapeutic value of phosphates, we are not disposed to fly in the face of authority, and question, but that the administration of this or any other salt of lime, in the absence of proper digestion, will aid in the elaboration of tooth structure in the fœtus or infant, we have not the least faith. And the food in ordinary use, we are sure contains an abundance of such materials for the ordinary wants of the system.

